# The Commencement in the University of Buffalo

**Published:** 1879-03

**Authors:** 


### Editorial.

### The Commencement in the University of Buffalo.

The exercises of the thirty-third annual Commencement of the Medical Department of the University of Buffalo were held at St. James Hall Tuesday evening February 25th, and was an occasion of great interest.

The annual meeting of the Council was held in the committee room of the Young Men's Association at four o'clock in the afternoon, the following members being present: The Hon. O. H. Marshall, President; Dr. James P. White, Dr. Thomas F. Rochester, Dr. Julius F. Miner, Dr. George B. Hayes, the Hon. Mayor Scheu, the Hon. E. G. Spaulding, the Hon. James O. Putnam, Mr. John Wilkeson, Mr. George S. Hazard, Mr. John D. Shepard, Mr. David Gray and Mr. James N. Matthews, Secretary.

Dr. White, as President of the Faculty reported that the Faculty and Curators had made the proper examinations and unanimously recommended that the degree in Doctor in Medicine be conferred upon each of the following gentlemen:

1. Edwin C. Taylor, Breeseport, N. Y. 2. W. H. Addington, Buffalo, N. Y. Charles Albert Wall, Buffalo, N. Y. 4. Andrew McKenny, Aylmer, Ont. 5. L. E. Ellis, Brocton, N. Y. 6. Frederick Jaekle, Dunkirk, N. Y. 7. Isaac Nelson Wright, Honeoye, N. Y. 8 Joseph B. Hatch, Rochester, N. Y. 9. Charles W. Shaver, Buffalo, N. Y. 10. W. L. Dickinson, West Webster, N. Y. 11. John B. La Mothe, Schediac, N. B. 12. Arthur A. Kendall, Corning, N. Y. 13. Chester Howard, Andover, N. Y 14. Samuel E. Ballard, Sacketts Harbor, N. Y. 15. Adelbert J. Clark, Wilson, N. Y. 16. F. F. Greene, Cazenovia, N. Y. 17. Floyd B. Parke, Breeseport, N Y. 18.

Frank H. Bartlett, Olean, N. Y. 19. Herbert Paris Sheldon, Perry, N. Y. 20. Jacob E. K. Morris, Olean, N. Y. 21. Robert W. Eastman, Owego, N. Y. 22. Charles Sumner Gere, Chemung, N. Y. 23. James Theophilus Stokes, Yates, N. Y. 24. Emory John Drury, Minetto, N. Y. 25. John H. Wiggins, Jamestown, N. Y. 26. Adelbert Eugene Crowell, Brookfield, N. Y. 27. Benj. F. Rogers, Albion, N. Y. 28. J. Watson Brown, Ithaca, N. Y. 29. Denton W. Rudgers, Pavilion, N. Y. 30. Geo. Albert Westfall, Union Centre, N. Y. 31. Tousley Lewis, Albion, N. Y. 32. Louis A. Bull, Buffalo, N. Y. 33. Henry Proper Stilwell, Mecklenburg, N. Y. 34. Ed. W. Trowbridge, Watertown, N. Y. 35. Eugene Orville Bardwell, Penn Yan, N. Y. 36. Edwin S. Miller, Mayville, N. Y. 37. David M. Bemus, Jamestown, N. Y. 38. Wilfred Hanland Sage, Hinsdale, N. Y. 39. Charles W. Robertson, Ransomville, N. N. 40. W. H. Pitt, Buffalo, N. Y. 41. John H. Thrall, Woodstock, Ont.

By vote of the Council, the honorary degree of Doctor in Medicine was conferred upon Professor Charles Avery Doremus, Ph. D., and Dr. Charles Cary was elected Professor of Anatomy.

The exercises of the evening were opened with prayer by Rev. Dr. Van Bokkelen, after which the President of the Faculty, Prof. Pames P. White, M. D., read the report of the Council of the University, including the list of graduates, and addressed the President of the Council, Hon. O. H. Marshall, as follows:

Mr. President—These candidates have shown, by proper certificates, that they have pursued the study of medicine under competent preceptors for the allotted time of three years; that they are of good moral character, and have reached the age of twenty-one years. They have given satisfactory evidence of their attendance upon two full courses of lectures at recognized Medical Colleges, the last course having been at the Medical Department of the University of Buffalo. They have written and submitted acceptable theses; passed examinations before the Faculty and Curators in all the departments taught in this college; and the Council of the University has formally directed that they receive the Degree of Doctor in Medicine. I therefore in behalf of the Faculty and Curators present them to you.

He also stated that Frederick Peterson had passed an excellent examination, but owing to the fact that he was not of age, a diploma could not be legally granted; and further that the thesis of W. H. Pitt upon "Spectrum analysis of the blood" deserved mention, and should be published.

The diplomas were then presented to the graduates by President Marshall, after which the charge to the graduates was delivered by Hon. E. Carlton Sprague, and was a scholarly, thoughtful, suggestive address, which was thoroughly appreciated by the Doctors, and by an intelligent audience, and was warmly received. Following this came the address before the Alumni Association delivered by Dr. Judson B. Andrews, Assistant Physician in the State Asylum for the Insane at Utica, upon the "Early Indications of insanity," which we shall publish in our next Journal.

